# Factors associated with intraoperative extracorporeal membrane oxygenation support during lung transplantation

**DOI:** 10.1186/s12931-020-01355-7

**Published:** 2020-04-15

**Authors:** Rong Zhang, Yonghao Xu, Ling Sang, Sibei Chen, Yongbo Huang, Lingbo Nong, Chun Yang, Xuesong Liu, Dongdong Liu, Yin Xi, Weiqun He, Bing Wei, Jianxing He, Yimin Li, Xiaoqing Liu

**Affiliations:** The State Key Laboratory of Respiratory Diseases, Guangzhou Institute of Respiratory Health, The First Affiliated Hospital of Guangzhou Medical University, 151 Yanjiang Street West, Guangzhou, 510120 Guangdong China

**Keywords:** Extracorporeal membrane oxygenation, Lung transplantation, Factors

## Abstract

**Background:**

Intraoperative Extracorporeal membrane oxygenation (ECMO) is increasingly being applied as life-support for lung transplantation patients. However, factors associated with this procedure in lung transplantation patients have not yet been characterized. The aim of this study was to identify preoperative factors of intraoperative ECMO support during lung transplantation and to evaluated the outcome of lung transplantation patients supported with ECMO.

**Methods:**

Patients underwent lung transplantation treated with and without ECMO in Guangzhou Institute of Respiratory Diseases between January 2015 to August 2018 were retrospectively reviewed. Patient demographics and clinical variables were collected and analyzed. Multivariate logistic regression was performed to identify factors independently associated with intraoperative extracorporeal membrane oxygenation support during lung transplantation.

**Results:**

During the study period, 138 patients underwent lung transplantation at our institution, the mean LAS was (56.63 ± 18.39) (range, 32.79 to 88.70). Fourty four patients were treated with veno-venous/veno-arterial ECMO. Among the patients, 32 patients wean successfully ECMO after operation, 12 patients remain ECMO after operation, and 32 patients (62.74%) survived to hospital discharge. In multiple analysis, the following factors were associated with intraoperative ECMO support: advanced age, high PAP before operation, duration of mechanical ventilation before operation, a higher APACHE II and primary diagnosis for transplantation. The overall survival rates at 1, 3, and 12 months were 90.91, 72.73, and 56.81% in the ECMO group, and 95.40, 82.76, and 73.56% in the non-ECMO group, respectively (log-rank *P* = 0.081). Patients who underwent single lung transplant had a lower survival rates in ECMO group as compared with non-ECMO group at 1, 3, and 12 months (90.47% vs 98.25, 71.43% vs 84.21, and 52.38% vs 75.44%) (log-rank *P* = 0.048).

**Conclusions:**

The preoperative factors of intraoperative ECMO support during lung transplantation included age, high PAP before operation, preoperative mechanical ventilation, a higher APACHE II and primary diagnosis for transplantation based on multivariate analysis.

## Background

Lung transplantation (LT) has rapidly become a valid therapeutic option for patients with end-stage lung disease. Since the indications for lung transplantation have broadened, the proportion of critically ill patients undergoing lung transplantation has grown considerably [1, 2]. The management of these LT patients has correspondingly become more complicated, and intraoperative cardiopulmonary support is often needed in these critically ill recipients [3].

Extracorporeal membrane oxygenation (ECMO) is a life-support technology device that performs gas exchange external to the body by providing cardiorespiratory support in patients with severe respiratory and cardiac failure [4]. Since the introduction of intraoperative ECMO to replace cardiopulmonary bypass (CPB) in 2001, most centers have switched to the routine use of ECMO intraoperative from 2008 on with favorable results [5–8]. Intraoperative ECMO is generally used in patients with pulmonary hypertension, hemodynamic instability, inability to tolerate single-lung ventilation, or hyper perfusion due to the reduced lung size during lung transplantation [3]. However, preoperative factors of this procedure in lung transplantation patients have not yet been characterized, and the decision to employ ECMO during lung transplantation depends largely on individual institutional practices [7–10]. Nevertheless, ECMO implantations without any a priori indication or inappropriate patient selection may increase resource utilization and hospital costs and may even be associated with significant morbidity and mortality [11, 12]. So early identification of the preoperative factors associated with the need for intraoperative ECMO is urgently needed. However, few retrospective reports have addressed this issue to date.

Our hospital is one of the most important medical centers for pulmonary diseases in mainland China. Based on promising initial experiences, ECMO has recently become our preferred mode of support for lung transplantation patients requiring cardiopulmonary support for intraoperative respiratory or hemodynamic instability. In this study, we hypothesized that there are preoperative variables may facilitate clinical decision making when considering patients for ECMO support during lung transplantation. In the current study, we analyzed the epidemiological characteristics and clinical features of lung transplantation patients in our center and evaluated the preoperative factors of intraoperative ECMO support.

## Materials and methods

### Study design and patients

The study was conducted at the First Affiliated Hospital of Guangzhou Medical University. The study was approved by the local research ethics committee (2018-K-14), which waived the need for informed consent for the retrospective collection of demographic and hospital outcome data based on Chinese legislation. Donor lungs were obtained from brain-dead organ donors via an organ procurement organization. One humdred thirty eight patients who underwent lung transplantation at our institution between January 2015 and August 2018were included in the study. Seven patients supported with ECMO as a bridge to lung transplantation were excluded. The remaining patients were divided into ECMO and non-ECMO groups. The protocol for evaluating the need for intraoperative ECMO are presented in Figure 4(supplement). Indication for intra-operative ECMO implant was set when the patients confronted with intraoperative hemodynamic instability (increased pulmonary pressure after pulmonary artery clamping), or severe hypoxemia (impaired gas exchange, hyper-perfusion, an inability to tolerate single lung ventilation) after optimization of patient cardiopulmonary conditions, a combination of the following conditions ensued: (1) hypercapnia (PaCO_2_>60 mmHg,PH < 7.2), (2) decrease of arterial saturation to < 90%, (3) cardiac index < 2 l/min/m^2^, cardiac index measured by Vigileo monitor (Edwards Lifesciences).

### Data collection

Basic information was collected from our institution’s database for all patients. The following retrospective data were obtained: age, sex, height and weight, body mass index (BMI), primary diagnosis for transplantation, preoperative mechanical ventilation time, preoperative ICU time, echocardiographic data before transplantation, blood gas values, lactate and serum creatinine, hemoglobin, platelet value, and APACHE II score, the lung allocation score (LAS). Operation type and duration, intraoperative blood product transfusion, ECMO mode, dates of hospitalization, discharge from the ICU, and complications were also recorded. Patient survival times were calculated from the date of surgery to the date of death or last contact.

### ECMO management

ECMO has been our preferred method of support since March 2012, and most patients were transplanted on intraoperative venoarterial ECMO. The ECMO implantation technique, circuits, and cannula types used at our institution have been reported elsewhere [13]. We used centrifugal pumps (Bioline, Maquet, Hirrlingen, Germany) at a flow rate of 3–5 L/min in all patients. Circuits were heparin-coated and composed of Quadrox PLS oxygenators (Bioline, Maquet, Hirrlingen, Germany) with HU 35 heater units (Maquet, Hirrlingen, Germany). Peripheral cannulation using the right femoral vein and artery was favored in cases with a minimally invasive approach using a limited anteroaxillary thoracotomy. Central cannulation in the ascending aorta and right atrium was used in cases with a standard clamshell incision or peripheral vascular disease. We used centrifugal pumps with a flow rate of 3–5 L/min. Anticoagulation was maintained by continuous intravenous unfractionated heparin targeting an activated clotting time of 160–180 s.

### Statistical analyses

Continuous variables were expressed as the mean ± standard deviation and categorical variables were expressed as percentages. Ordinal variables were presented as median (interquartile range). The means of continuous variables and normally distributed data were compared using Student’s t-test. Categorical data were tested using the χ2 test. Survival estimates were calculated by the Kaplan–Meier product-limit method. Logistic regression analysis was used to assess factors for ECMO support, with results reported as odds ratios and 95% confidence intervals. *P* <  0.05 was considered statistically significant. Statistical analysis was performed using SPSS 19.0.

## Results

### Patient characteristics

**Fig. 1:**
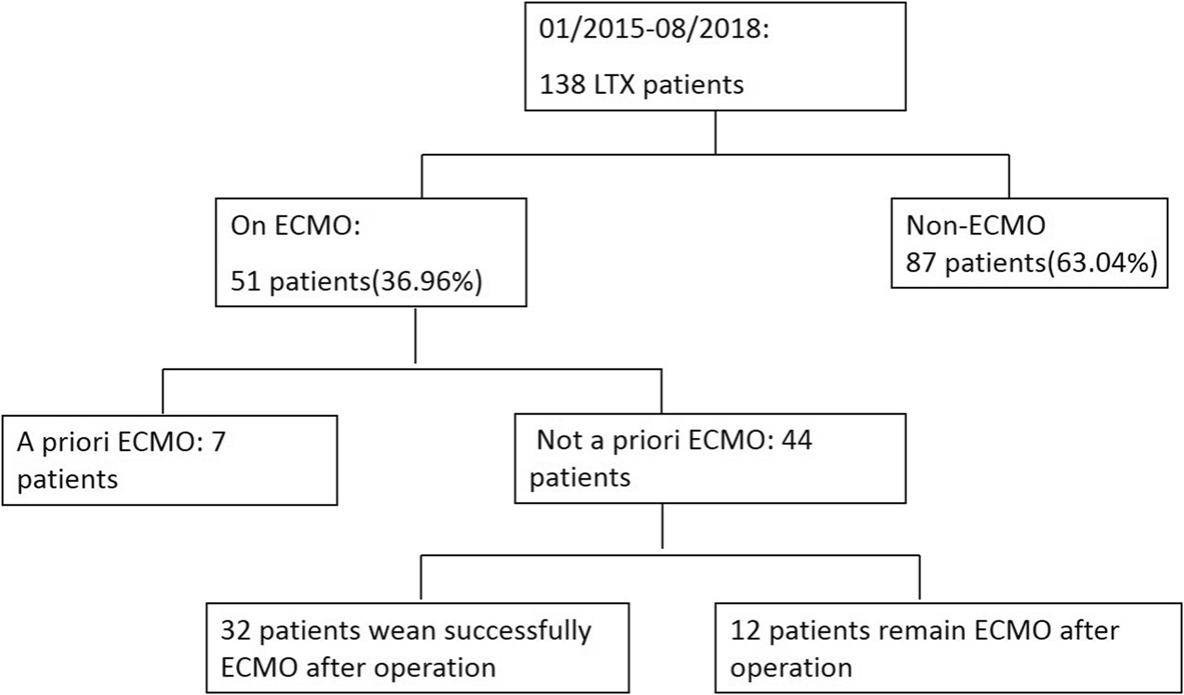
Flowchart of patient groups in the study

One thirty eight lung transplantation recipients were analyzed during the study period, of whom 87 (63.04%) underwent transplantation without intraoperative ECMO support and the remaining 51 (36.96%) required ECMO during lung transplantation. Seven patients supported with ECMO as a bridge to lung transplantation were excluded from the study. Thirty-two patients were weaned successfully off ECMO after surgery and the other twelve showed a complicated postoperative course requiring prolonged postoperative ECMO support (Fig. 1). The preoperative patient characteristics are summarized in Table 1. One hundred eighteen patients were male, and the mean age was 57.85 ± 12.03 years (range 27–75 years). The average age of the ECMO group was significantly higher than that of the non-ECMO group (56.28 ± 13.02 vs 62.70 ± 6.21 years, *P* <  0.05). The mean BMIs in the two groups were similar (19.26 ± 5.76 vs 19.59 ± 3.11 kg/m2, *P* = 0.77). The primary diagnosis for lung transplantation among the patients were lung fibrosis (*n* = 77), chronic obstructive pulmonary disease (COPD) (*n* = 47), bronchiectasis (*n* = 5) and re-transplantation (*n* = 2). It was noted that the ECMO group was more used in patients diagnosed with idiopathic pulmonary fibrosis (IPF) (22.73% versus 6.90%, *p* = 0.02) and re-transplant but less used in patients with COPD (13.64% versus 47.13%, *p* <  0.01) compared with the non-ECMO group. The APACHE II score was significantly higher in the ECMO group compared with the non-ECMO group (22.48 ± 1.87 vs 15.28 ± 7.08, *P* <  0.05). The mean LAS was (56.63 ± 18.39) (range, 32.79 to 88.70). Patients supported by ECMO tended to have higher LAS but there was no significant difference in the LAS between the two groups (61.89 ± 18.63 vs 55.85 ± 15.22, *P* = 0.056). Donor characteristics in the two groups are shown in Table 2. All donors were brain-dead individuals, with a mean age of 34.00 ± 12.11 years. Demographic data of lung donor were similar between groups in terms of age. Donors in the ECMO group tended to have longer periods of ventilation and travel time, though the difference was not significant. There is an increasing need for intraoperative blood transfusions in the ECMO group (12.94 ± 8.64 units, vs 6.78 ± 5.05 units, *P* <  0.01). Among the 131 recipients, 53 patients underwent sequential double lung transplantation and 78patients received single lung transplantation. Recipients who required ECMO had significantly longer periods of mechanical ventilation and ICU stays before operation (6.38 ± 9.04 vs 1.37 ± 4.69 days, *P* <  0.05, 7.67 ± 9.12 vs 2.60 ± 7.23 days, respectively; *P* <  0.05) than patients in the non-ECMO group. Preoperative echocardiography was performed in lung transplantation patients, and the PAP lever was higher in the ECMO group compared with the non-ECMO group (50.83 ± 1.20 versus 40.58 ± 14.84 mmHg, *p* <  0.05). ECMO patients had more severe respiratory failure compared with the non-ECMO group, with a mean preoperative PaO2/FiO2 of 75.93 ± 31.74 mmHg (Table 3). Lactate and lymphocytes were significantly higher in the ECMO compared with the non-ECMO group after operation (5.97 ± 3.62 vs 4.24 ± 2.52, *P* = 0.031, 0.57 ± 0.42 vs 0.38 ± 0.22, *P* = 0.020). Notably, serum creatinine was significantly increased in both groups after surgery, with a greater increase in the ECMO group.

**Table 1:** Baseline characteristics of lung transplant recipients with and without ECMO

Characteristic	Total(***n*** = 131)	ECMO(***n*** = 44)	non-ECMO (***n*** = 87)	***P*** -value
Age (years)	57.85 ± 12.03	62.70 ± 6.21	56.28 ± 13.02	0.04
Sex (male/female)	118/13	38/6	80/7	
BMI (kg/m^2^)	19.48 ± 4.08	19.26 ± 5.76	19.59 ± 3.11	0.77
Transplant indication				
Lung fibrosis	77	33	44	0.009
IPF	16	10	6	0.02
NSIP	29	12	17	0.52
Pneumosilicosis	7	3	4	0.69
CTD-fibrosis	15	3	12	0.30
Other	10	5	5	0.30
COPD	47	6	41	< 0.01
Re-transplant	2	2	0	0.045
Bronchiectasis hemoptysis	5	3	2	0.33
Co-morbidities				
Hypertension	18	5	13	0.79
Diabetes mellitus	19	10	9	0.07
Barotrauma	11	5	6	0.51
LAS	58.63 ± 18.39	61.89 ± 18.63	55.85 ± 15.22	0.056
APACHE II score	17.54 ± 8.23	22.48 ± 1.87	15.28 ± 7.08	< 0.01

**Table 2:** Data for lung transplant recipients and donors comparing ECMO and non-ECMO

Characteristic	Total(***n*** = 131)	ECMO(***n*** = 44)	non-ECMO (***n*** = 87)	***P*** value
Operative time	7.03 ± 2.71	8.74 ± 2.32	6.09 ± 2.45	< 0.01
Single lung	78	21	57	0.06
Bilateral lung	53	23	30	0.06
Lung volume reduction	4	0	4	0.30
Intraoperative transfusion	8.52 ± 9.25	12.94 ± 8.64	6.78 ± 5.05	< 0.01
Preoperative mechanical ventilation	5.08 ± 9.00	6.38 ± 9.04	1.37 ± 4.69	0.01
Preoperative intensive care unit	2.76 ± 6.61	7.67 ± 9.12	2.60 ± 7.23	0.02
PAP (mmHg)	44.67 ± 13.08	50.83 ± 1.20	40.58 ± 14.84	0.01
Donor data				
Age (years)	34.00 ± 12.11	35.00 ± 16.58	32.54 ± 10.84	0.52
Travel time(h)	4.62 ± 2.31	5.24 ± 4.12	4.29 ± 3.15	0.058
Ventilation (days)	4.19 ± 5.77	4.94 ± 6.83	2.44 ± 1.54	0.14

**Table 3:** Data of lung transplant recipients comparing ECMO and non-ECMO

Characteristic		Total(***n*** = 131)	ECMO(***n*** = 44)	non-ECMO (***n*** = 87)	***P*** value
PaO_2_/FiO_2_	Before	83.68 ± 45.99	75.93 ± 31.74	87.13 ± 51.01	0.05
	After	175.10 ± 85.64	192.40 ± 103.5	167.40 ± 76.45	0.28
PaCO_2_	Before	55.74 ± 13.75	56.30 ± 14.88	55.38 ± 13.14	0.78
	After	43.73 ± 10.25	37.30 ± 10.14	47.81 ± 8.05	< 0.01
WBC count, 10^9^/L	Before	9.03 ± 4.55	10.33 ± 6.02	8.57 ± 3.72	0.16
	After	13.93 ± 10.28	11.99 ± 7.37	15.01 ± 11.49	0.29
Lymphocytes	Before	0.87 ± 0.66	0.92 ± 0.72	0.84 ± 0.65	0.65
	After	0.43 ± 0.30	0.57 ± 0.42	0.38 ± 0.22	0.02
Hemoglobin	Before	107.40 ± 22.75	98.00 ± 16.75	108.80 ± 18.63	0.03
	After	118.3 ± 23.07	102.60 ± 16.39	124.90 ± 22.75	< 0.01
Lactate, mmol/L	Before	1.12 ± 0.96	1.26 ± 0.86	1.06 ± 1.02	0.47
	After	4.78 ± 2.99	5.97 ± 3.62	4.24 ± 2.52	0.03
PLT, 10^9^/L	Before	193.70 ± 88.49	182.30 ± 95.36	200.60 ± 87.26	0.45
	After	129.10 ± 60.20	106.20 ± 54.05	140.70 ± 61.32	0.04
Serum creatinine	Before	60.95 ± 23.45	70.30 ± 28.36	56.72 ± 19.94	0.03
	After24h	96.88 ± 50.56	124.60 ± 57.08	81.48 ± 37.48	< 0.01
	After48h	85.17 ± 51.47	122.00 ± 56.42	65.93 ± 32.68	< 0.01

### Patient outcomes and statistical analyses

**Fig. 2:**
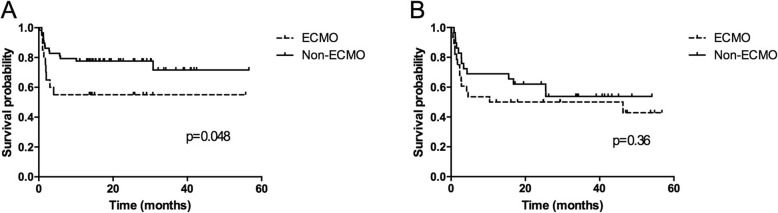
Kaplan–Meier survival functions in single (**a**) and bilateral (**b**) lung transplant recipients with and without ECMO

**Fig. 3:**
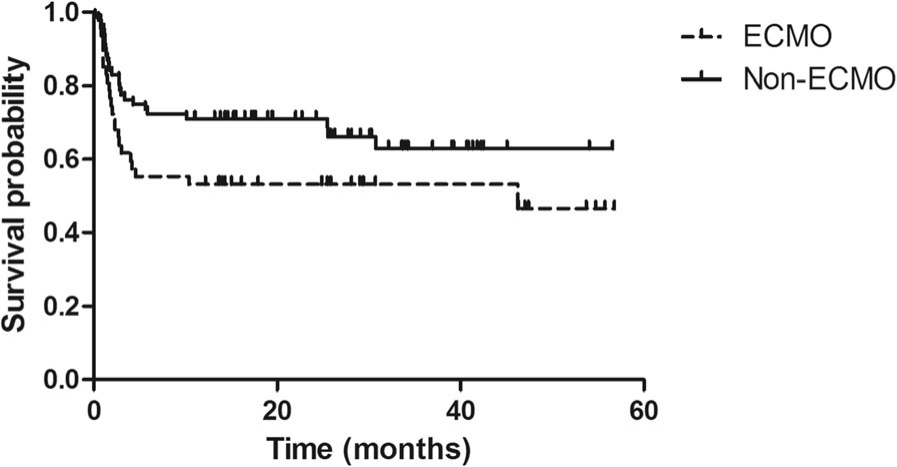
Overall survival in lung transplant recipients with and without ECMO

The complications and outcomes in lung transplantation recipients with and without ECMO are listed in Table 4. Hemorrhagic events occurred in four patients in ECMO group compared with two in the non-ECMO group, though the difference was not significant (*P* = 0.09). Patients in the ECMO group had a significantly higher rate of renal failure requiring dialysis compared with the non-ECMO group (52.27% vs 9.20%, *P* <  0.001). With respect to early post-transplant outcomes, the median length of ICU stays for patients supported on ECMO was 34.52 ± 35.02 days (range, 10–70 days) compared with 20.09 ± 21.56 days (range, 6–41 days) in the non-ECMO group. The patients who underwent bilateral lung transplant had a 1-, 3 and 12-month survival rates of 91.30, 73.91, 60.87% in ECMO group, and 90, 80, 70% in non-ECMO group respectively (log-rank *P* = 0.36). For single lung transplantation patients, survival rate at 1, 3, and 12 months were 90.47, 71.43, 52.38% in ECMO group, and 98.25, 84.21, 75.44% in non-ECMO group, respectively (log-rank *P* = 0.048) (Fig. 2). The overall 1-, 3-, and 12-month survival rates were 90.91, 72.73, 56.81% in the ECMO group and 95.40, 82.76, 73.56% in the non-ECMO group. Analysis of overall survival among patients after transplant revealed there are no differences in survival between the ECMO and non-ECMO cohorts (Fig. 3) (log-rank *P* = 0.081).

**Table 4:** Complications and clinical outcomes of lung transplant recipients comparing ECMO and non-ECMO

Complications	Total(***n*** = 131)	ECMO(***n*** = 44)	non-ECMO (***n*** = 87)	***P*** value
Bleed	6	4	2	0.09
Renal failure	15	11	4	< 0.01
Ventilation after operation	18.97 ± 32.51	25.21 ± 38.50	17.22 ± 29.13	0.37
Length of ICU stay, days	24.75 ± 27.24	34.52 ± 35.02	20.09 ± 21.56	0.05
Length ofhospital stay, days	53.81 ± 33.71	55.27 ± 34.95	53.14 ± 33.54	0.81
ICU mortality n(%)				
1 month	8 (6.10%)	4 (9.09%)	4 (4.60%)	0.44
3 month	27 (20.61%)	12 (27.27%)	15 (17.24%)	0.25
12 month	42 (32.06%)	19 (43.18%)	23 (26.44%)	0.07

Multivariate logistic regression was performed using factors that achieved a level of significance of less than 0.05 in the bivariate analysis (Table 5). Briefly, recipient age, duration of mechanical ventilation and ICU stays, PAP level, PaO_2_/FiO_2,_ hemoglobin, serum creatinine before operation, APACHE II and underlying lung disease correlated with intraoperative ECMO support. In multiple analysis, the following factors were associated with intraoperative ECMO support: advanced age, high PAP before operation, duration of mechanical ventilation before operation, a higher APACHE II and primary diagnosis for transplantation.

**Table 5:** Multivariate logistic regression analysis of independent predictors of ECMO support

	Univariate logistic regression		Multivariate logistic regression		
Variables	OR	*p*	OR	95%CI	*p*
Age > 60	14.20	0.01	11.86	1.422–99.08	0.02
Preoperative mechanical ventilation	1.11	0.01	49.45	2.85–858.75	0.01
Preoperative intensive care unit	1.07	0.03			
PAP before operation	1.07	0.01	0.91	0.83–1.01	0.05
Preoperative APACHE II	1.15	0.01	46.21	3.22–662.38	0.01
Preoperative PaO_2_/FiO_2_	0.98	0.05			
Preoperative hemoglobin	0.97	0.04			
Preoperative serum creatinine	1.02	0.03			
Underlying lung disease	0.02	0.01	0.02	0.001–0.32	0.01

## Discussion

Evolution in technology has resulted in rapid increase in utilization of ECMO in lung transplantation. Intraoperatively, ECMO helps to overcome excessive pulmonary hypertension and associated right heart failure after clamping of the pulmonary hilum or global hypoxia and hypercarbia during one lung ventilation [5]. However, few studies have reported to evaluate the preoperative factors of intraoperative ECMO during lung transplantation. Our results identified age, duration of ventilation before operation, PAP levels, primary diagnosis for transplantation, and preoperative APACHE II score as factors associated with intraoperative ECMO support in lung transplantation patients.

To the best of our knowledge, studies evaluating factors of intraoperative ECMO during lung transplantation are limited. Anastasios et al. failed to identify any preoperative predictors of the need for bypass [14], while de Hoyos and colleagues reported on the Toronto experience and indicated that preoperative room air oxygen tension, pulmonary hemodynamics, oxygen requirements, right ventricular function, and exercise capacity could be used as predictors for bypass requirements in patients undergoing lung transplantation [15]. In the present study, we observed that the intraoperative ECMO support was more likely to be required in older recipients. Age has been a well-known prognostic factor for lung transplantation and several studies have shown that age was significantly correlated with ECMO support in other disease [16–18]. In addition to age, we found that the duration of mechanical ventilation before operation and higher preoperative APACHE II scores were also associated with ECMO support during lung transplantation, which suggests that patients requiring pre-transplant mechanical ventilation and had higher preoperative APACHE II scores should be considered for ECMO support. Moreover, we confirmed that recipients who required ECMO support during lung transplantation tended to have higher PAP levels, which was also in accord with other reports [19, 20]. Patients with severe pulmonary hypertension often have significant right ventricular dysfunction, and decreased cardiac output, hemodynamic instability, and global lung dysfunction may be counterbalanced by venoarterial and venovenous ECMO systems. Notably, the current study found that the type of end-stage lung disease was an important factor influencing the use of intraoperative ECMO in lung transplantation patients. In our study, IPF was a common indication for lung transplantation and 10/44 patients at our institution with severe fibrosis and all re-transplant patients were transplanted on ECMO support, compared with COPD patients. This suggests that patients with underlying lung diseases such as fibrosis and thus undergoing re-transplant should be recommended to undergo ECMO. Other factors such as a longer ICU stay before transplantation, lower PaO2/FiO2 ratio, lower hemoglobin and higher serum creatinine measured before operation was associated with intraoperative ECMO support in univariate regression analysis, but these variables were not independently associated with intraoperative ECMO support by multivariate logistic regression analysis.

The current results showed that ECMO was employed in 33.59% of lung transplantation patients. Although patients in the ECMO group had a more complicated course than non-ECMO patients, there was no significant difference between the groups in terms of in-hospital mortality, with 1-, 3-, and 12-month survival rates of 90.91, 72.73, 56.81% in the ECMO group and 95.40, 82.76, 73.56% in the non-ECMO group. Bermudez et al. assessed survival in 49 patients who underwent LT with ECMO support between 2007 and 2013, the survival rates at 1, 6, and 12 months were 95.9, 85.7, and 80.9% in the ECMO group and 97.6, 91.7, and 86.1% in the non-ECMO group [21]. The Munich lung group also published their experience with intraoperative ECMO application, they reported a perioperative mortality of 11.1% and a 1-year survival of 81.5% in the ECMO group and 4.5, and 81.8% in the non-ECMO group [9]. The Vienna group summarized their experiences of intraoperative ECMO implementation in bilateral lung transplantation between 2010 and 2016, and reported a 1-year survival rate of 91% in the ECMO group 82% in the non-ECMO group [22]. Ko et al. demonstrate the survival rate in single lung transplantation undergoing ECMO was 66.7% [23]. Compared with previous studies, the present study reported a relatively higher mortality rate in lung transplantation patients with ECMO support. This could be attributable to several factors. First, the patients enrolled in our study were seriously ill as indicated by high LAS. The median LAS score was 58.63 ± 18.3(32.79–88.0),which was much higher that other centers especially in the ECMO group [24]. There is approximately 20.61% (27/131) of patients needed mechanical ventilation pre-transplantation, and the mortality of these patients would thus be expected to be significantly higher than that of other lung transplantation patients. Secondly, in our study it should be noted that the survival rate was significantly worse for single lung transplantation recipients undergoing ECMO, which may also have contributed to poor overall outcomes. It might probably due to excessive bleeding and transfusion requirements and prolonged ischemic time during transplantation. Finally, other groups have recently reported on the use of ECMO support profilactically in lung transplantation showed an improve survival, which could demonstrate the benefit of prophylactic ECMO [25, 26]. However, in our center ECMO was usually only reserved for complex transplantation or to support intraoperative unstable patients, which may also influence the outcome. And the mortality rate is likely to be strongly influenced by criteria that vary among centers, and regional differences in organ availability, institutional preferences related to the use and management of ECMO, and surgeon-specific preferences regarding organ selection and operative techniques may all have profound effects on the outcomes of ECMO during lung transplantation [24].

Some limitations of this study should be acknowledged. First, the lung transplantation patient population in the current study is relatively small. It is not possible to include abundant study subjects due to the donor lung organ insufficiency. Secondly, it was a retrospective study performed at a single medical center, which thus limited the generalizability of the findings. Third, unmeasured variables such as infiltration in radiography, extent of atelectasis, microbiologic data, and intraoperative events may have influenced the results.

## Conclusions

In conclusion, the results of the current study demonstrate that advanced age, long duration of ventilation before operation, high PAP, underlying lung disease, and APACHE II score before operation may influence the likelihood of requiring intraoperative ECMO during lung transplantation. It may help physicians in decision making processes and optimize the allocation of resources.

## Supplementary information

**Additional file 1 Figure 4**. Process reports intraoperative ECMO support in LTX patients. PA (pulmonary artery); LTX (lung transplantation).

## Data Availability

The datasets analyzed during the current study are not publicly available, but are available from the corresponding author on reasonable request.

## Abbreviations

ECMO: Extracorporeal membrane oxygenation
LT: Lung transplantation
PAP: Pulmonary arterial pressure
APACHE II: Acute physiology and chronic health evaluation II
CPB: Cardiopulmonary bypass
ICU: Intensive care unit
BMI: Body mass index
VAECMO: Venoarterial ECMO; IPF: idiopathic pulmonary fibrosis
COPD: Chronic obstructive pulmonary disease
NSIP: Nonspecific interstitial pneumonia
CTD: Connective tissue diseases
LAS: Lung allocation score
