# Supplementary material for: Factors associated with intraoperative extracorporeal membrane oxygenation support during lung transplantation
Source: Respir Res. 2020 Apr 15;21:85. doi: 10.1186/s12931-020-01355-7 (PMC7160893; doi:10.1186/s12931-020-01355-7)
Supplement: Supplementary file 1 — Additional file 1 Figure 4. Process reports intraoperative ECMO support in LTX patients. PA (pulmonary artery); LTX (lung transplantation). [file 12931_2020_1355_MOESM1_ESM.docx]

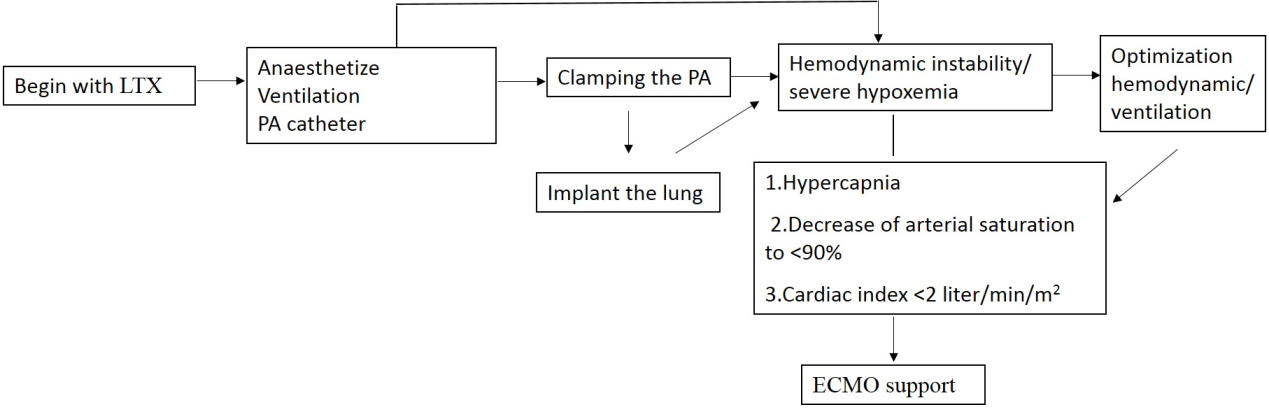


**Figure 4**. Process reports intraoperative ECMO support in LTX patients. PA (pulmonary artery); LTX (lung transplantation).
